# Protocol for tissue processing and paraffin embedding of mouse brains following *ex vivo* MRI

**DOI:** 10.1016/j.xpro.2023.102681

**Published:** 2023-11-09

**Authors:** Adele Smart, Cristiana Tisca, Istvan N. Huszar, Daniel Kor, Olaf Ansorge, Mohamed Tachrount, Sean Smart, Jason P. Lerch, Karla L. Miller, Aurea B. Martins-Bach

**Affiliations:** 1 Neuropathology, Nuffield Department of Clinical Neurosciences, University of Oxford, OX3 9DU Oxford, UK; 2 Wellcome Centre for Integrative Neuroimaging, FMRIB, Nuffield Department of Clinical Neurosciences, University of Oxford, OX3 9DU Oxford, UK

**Keywords:** Microscopy, Model Organisms, Neuroscience

## Abstract

Combining histology and *ex vivo* MRI from the same mouse brain is a powerful way to study brain microstructure. Mouse brains prepared for *ex vivo* MRI are often kept in storage solution for months, potentially becoming brittle and showing reduced antigenicity. Here, we describe a protocol for mouse brain dissection, tissue processing, paraffin embedding, sectioning, and staining. We then detail registration of histology to *ex vivo* MRI data from the same sample and extraction of quantitative histological measurements.

## Highlights

• Tissue processing optimized for mouse brains after *ex vivo* MRI and prolonged storage
• Details for sectioning difficult formalin-fixed paraffin-embedded samples
• Protocol for immunohistochemistry of mouse brain for different antibodies
• Registration of histology to *ex vivo* MRI data from the same mouse brain

## Abstract (editor highlights)

Publisher’s note: Undertaking any experimental protocol requires adherence to local institutional guidelines for laboratory safety and ethics.

## Graphical abstract

**Figure:**
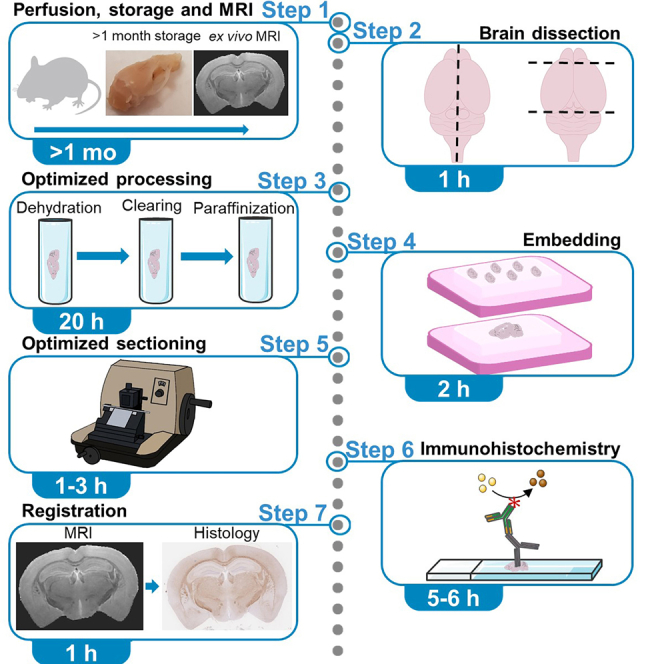

## Before you begin

A guideline for the following steps is provided in this protocol: 1) Perfusion, storage, and *ex vivo* MRI, 2) Brain dissection, 3) Processing of tissue prepared for MRI, 4) Embedding into paraffin blocks, 5) Sectioning, 6) Immunohistochemistry, and 7) Registration of histological images to MRI.

Before starting the protocol, animals must have been fixed with intracardiac perfusion, brains collected and imaged with MRI. This guideline is optimized for adult mouse brain tissue that was stored for an extended time for *ex vivo* MRI and describes how to embed both single and multiple mouse brain samples within a single block and how to get optimized histological slides that can then be registered to MRI.

### Institutional permissions

Samples used in this study were collected by collaborators in the European Union or the United Kingdom. Perfusions were performed following the guidelines of the European (2010/63/EU) or UK Home Office legislation (Animals Scientific Procedures Act 1986), with the approval of the relevant ethics committees and/or the United Kingdom Home Office. Institutional approval was not required for experiments involving *ex vivo* mouse tissue.

### Perfusion

**Timing: 2–3 h**

For a detailed procedure refer to Spring et al., 2007^1^ or Cahill et al., 2012.^2^ In this protocol we used mouse brains obtained from the mouse models listed in the key resources table.

1. Set up the perfusion pump, absorbent mats, and equipment at a perfusion table.
2. Anesthetize the mice with Ketamine (150 mg/kg) and Xylazine (10 mg/kg).
  a. Mix with saline solution to have 0.1 mL solution per 10 *g* body weight. Administer via intraperitoneal injection.

***Alternatives:*** Other anesthetizing agents can be used. Abide by your institutional protocol for euthanizing mice when performing cardiac perfusions.

3. Open the chest cavity and clamp the xiphoid process with a hemostat.
4. Insert a butterfly needle into the left ventricle of the heart for transcardiac perfusion and cut the right auricle to allow the blood to escape.

***Note:*** Some researchers insert a blunt needle into the ascending aorta helps to prevent passing a sharp needle through the heart and maximize perfusion of the carotid arteries.

5. Turn on the pump and perfuse the mouse with 30 mL of room temperature perfusion solution.
  a. Perfusion solution 1: 1X PBS (Phosphate Buffered Saline, Sodium chloride: 0.137 M, Potassium chloride: 2.7 mM), 1 μL/mL heparin and 2 mM Gadovist at a flow rate of 1.0 mL/min.
6. Add 30 mL of perfusion solution 2 when there is almost no perfusion solution 1 left in the channel.
  a. Perfusion solution 2: 4% buffered paraformaldehyde (PFA: prepared by mixing one 10 mL vial of 16% pre-made PFA 10X with 30 mL of 1X PBS) and 2 mM Gadovist at a flow rate of 1.0 mL/min.
  b. Avoid air entering the channel.

**CRITICAL:** Paraformaldehyde (PFA) is hazardous and should be handled at the vented perfusion table with down draft or in a fume hood. Wear protective gloves, protective clothing and safety glasses. Abide by your institutional protocols when using and discarding PFA.

7. After perfusion, remove the head, trim skin, ears, lower jaw, and muscle tissues but keep the brain inside the skull.
8. Place the brain in the skull in perfusion solution 2 overnight (12 h) at 4°C.
  a. Perfusion solution 2: 4% PFA (prepared by mixing one 10 mL vial of 16% pre-made PFA 10X with 30 mL of 1X PBS) and 2 mM Gadovist.
9. The next day, transfer the sample to storage solution and store in the fridge at 4°C.
  a. Storage solution: 1 X PBS, 0.02% sodium azide, 2 mM Gadovist.
  b. Store the samples in this solution for at least one month before performing *ex vivo* MRI.^3^ Some of the samples we used in this protocol were stored for up to 27 months.

**CRITICAL:** Samples must not be frozen at any point during this protocol as it will affect the tissue microstructure and create artefacts in both MRI and histology.

**CRITICAL:** Sodium azide is toxic and should be handled in a chemical fume hood. Wear protective gloves, protective clothing and safety glasses.

***Alternatives:*** ProHance (Bracco) can be used as an alternative to Gadovist. Volumes need to be adjusted since ProHance is sold at 0.5 M while Gadovist is available at 1 M.

### *Ex vivo* MRI

**Timing: 14 h**

For a complete protocol see the study by Tisca et al. 2021,^4^ Tisca et al. 2022.^5^ In our study, MRI was performed on a 7.0 T MRI scanner using a receive-only 4-channels surface cryoprobe and a volume transmit resonator (Bruker Biosystems, Ettlingen, Germany). Different scanners and probes could be used, in which case adjustments to the MRI protocols might be necessary.

**Figure 1:**
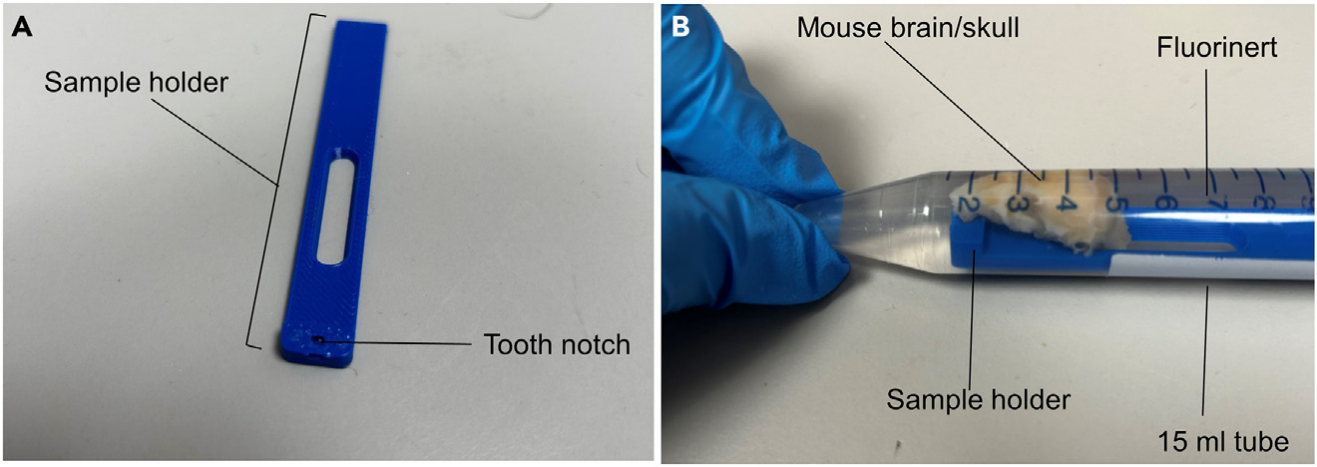
Image of the custom sample holder for *ex vivo* imaging (A) 3D printed sample holder. (B) The mouse skull is placed on the sample holder using the tooth notch. The holder is inserted into a 15 mL tube and filled with Fluorinert.
  d. Vacuum pump samples for up to 1 h (pressure 10–20 mbar to remove bubbles, in particular intracranial bubbles).
  e. Place the tube with the sample in the cryoprobe cradle or custom holder and slide into the cryoprobe, already positioned inside the scanner.

10. Prepare the samples for imaging.
  a. Remove the sample from the storage solution (PBS, azide and contrast agent).
  b. Remove any residue of PBS on the sample by gently wiping with a clean tissue.
  c. Place the sample into a 15 mL ultracentrifuge tube filled with Fluorinert (Figure 1B). We used a 3D printed holder to keep the sample in place and at the right orientation inside the 15 mL tube (Figure 1A).

***Alternatives:*** Fomblin LC/8 or Galdin Ht200 could be used as alternatives to Fluorinert.

11. Acquire the desired MRI modalities. In this study, we acquired structural MRI (60 μm isotropic resolution), diffusion-weighted MRI (100 μm isotropic resolution), and multi-echo gradient-echo data for QSM/T2∗ (100 μm isotropic resolution) using the parameters listed in Table 1.
  a. Keep the samples at room temperature (18°C–22°C) during acquisition.

**Table 1:** Acquisition details for T2-weighted, T2∗-weighted and diffusion-weighted MRI (dMRI)

MRI modality	Acquisition time	Resolution/Matrix size	Acquisition parameters
T2-weighted	33 min	60 × 60 × 60 μm/400 × 160 × 200	TurboRARE, TE = 12 ms, echo spacing = 12 ms, 6 echoes, TR = 350 ms, BW = 60 kHz
T2∗-weighted	26 min	100 × 100 × 100 μm/240 × 96 × 120	3D Multi-echo GRE, FA = 15°, TE1 = 3 ms, echo spacing = 3 ms, 20 echoes, TR = 68 ms, NR = 2, BW = 250 kHz
dMRI	13 h	100 × 100 × 100 μm/240 × 96 × 120	Segmented EPI, 12 segments, TE = 30 ms, TR = 500 ms, δ/Δ = 6.7/13.5 ms, b = 0 s/mm^2^ (volumes: 4 + 1 phase-encoding reversed), b = 2500 s/mm^2^ (volumes: 30), b = 10000 s/mm^2^ (volumes: 30), BW = 250 kHz, directions covering the whole shell and staggered between shells

Turbo Rapid Acquisition with Relaxation Enhancement (RARE), echo time (TE), repetition time (TR), bandwidth (BW), gradient echo (GRE), flip angle (FA), number of repetitions (NR), echo-planar imaging (EPI), duration of the diffusion-encoding gradient (δ), time interval between the two diffusion-encoding gradients (Δ), and b-value (b).
12. Once the scan is complete, gently remove the sample from Fluorinert.
  a. Wipe it with a clean tissue and place it back in storage solution.
  b. Keep the samples in the storage solution at 4°C until preparation for histology.

***Note:*** Keeping the samples in the storage solution containing 2 mM Gadovist ensures that MRI can be repeated if needed. In our case, the interval between sample collection and histology was up to 27 months.

## Key resources table

**Table:** 

REAGENT or RESOURCE	SOURCE	IDENTIFIER
**Antibodies**		

Goat anti-rabbit secondary, HRP, ready to use	Agilent Technologies	Cat# K400311-2
Rabbit anti-brevican (BCAN), 1:2,000	Abcam	Cat# ab285162
Rabbit anti-CD68, 1:400	Abcam	Cat# ab125212; RRID: AB_10975465
Rabbit anti-ferritin (FTH1), 1:300	Cell Signaling Technology	Cat# 4393; RRID: AB_11217441
Rabbit anti-myelin proteolipid protein (PLP), 1:500	Abcam	Cat# ab28486; RRID: AB_776593
Rabbit anti-NeuN, 1:3,000	Abcam	Cat# ab177487; RRID: AB_2532109
Rabbit anti-neurofilament 200 (NF), 1:2,000	Sigma-Aldrich	Cat# N4142; RRID: AB_477272

**Chemicals, peptides, and recombinant proteins**		

Antibody diluent, background reducing	Agilent Technologies	Cat# S302283-2
Citroclear	Genta Medical	Cat# CITO50
DPX	Sigma-Aldrich	Cat# 1005790500
Ethanol absolute	Sigma-Aldrich	Cat# 32221-M
Fluorinert	Acota	Cat# FC-770
Gadovist (gadolinium-based contrast agent)	Bayer	Cat# 30351004
Harris hematoxylin	Leica Biosystems	Cat# 3801560E
Heparin	Wockhardt	Cat# 30170145
Histoplast PE paraffin wax	SLS Ltd.	Cat# HIS3324
Hydrochloric acid 37% (HCl)	VWR	Cat# 20252.244
Hydrogen peroxide 30% (H_2_O_2_)	Fisher Scientific	Cat# 10687022
Industrial denatured alcohol (IDA)	Genta Medical	Cat# I99050
Ketamidor (ketamine) 100 mg/mL solution for injection	Chanelle Pharma	Cat# 30395940
Mollifex	VWR	Cat# 360584X
Oxoid phosphate-buffered saline (PBS) tablets	Thermo Scientific	Cat# BR0014g
Paraformaldehyde (PFA)	Electron Microscopy Sciences	Cat# 15710
Protein block, serum free	Agilent Technologies	Cat# X090930-2
Sodium azide	Sigma-Aldrich	Cat# S2002
Sodium chloride	Fisher Scientific	Cat# 10316943
Sodium hydroxide 2 mol/L (2 N) in aqueous solution (NaOH)	VWR	Cat# 98108.290
Tri-sodium citrate dihydrate (citrate)	Fisher Scientific	Cat# 10396430
Tris (hydroxymethyl) methylamine (Tris buffer)	Fisher Scientific	Cat# 10785341
Triton X	Sigma-Aldrich	Cat# X100
Xylazine	Bayer	Cat# 2150569
Xylene	Genta Medical	Cat# XYL050

**Critical commercial assays**		

Liquid DAB+ Substrate Chromogen System Kit	Agilent Technologies	Cat# 346811-2

**Experimental models: Organisms/strains**		

Mouse, Bcan knockout (BCAN-DEL465-EM1-B6N-IC) and wild-type littermates, 10 weeks, males and females	MRC Harwell	N/A
Mouse, FUSΔ14/Δ14 and wild-type littermates (C57BL/6J-DBA/2J genetic background), 12 weeks old, females	MRC Harwell^6^	N/A
Mouse, SOD1-G93A, 18 weeks, males and females	MRC Harwell	N/A
Mouse, Tardp-M323K and wild-type littermates, 12 months old, females	MRC Harwell^7^	N/A
Mouse, Vcan point mutation (VCAN-E441A-EM2-B6-IC) and wild-type littermates, 10 weeks, males and females	MRC Harwell	N/A

**Software and algorithms**		

Fiji ImageJ	National Institutes of Health^8^	RRID: SCR_003070 https://imagej.net/software/fiji/
FMRIB Linear Image Registration Tool (FLIRT)	University of Oxford	https://fsl.fmrib.ox.ac.uk/fsl/fslwiki/FLIRT
FMRIB Software Library (FSL)	University of Oxford^9^	RRID: SCR_002823 https://fsl.fmrib.ox.ac.uk/fsl/fslwiki/
FSLeyes	University of Oxford	https://git.fmrib.ox.ac.uk/fsl/fsleyes/fsleyes
Ihcpy (SAF; stained area fraction maps)	University of Oxford	https://git.fmrib.ox.ac.uk/spet4877/ihcpy/-/tree/main/
TIRL (version 3)	University of Oxford	https://git.fmrib.ox.ac.uk/ihuszar/tirl/-/tree/v3
TIRLscripts	University of Oxford	https://git.fmrib.ox.ac.uk/ihuszar/tirlscripts/-/tree/master/tirlscripts/oxford/mouse

**Other**		

Automatic tissue processor	Thermo Scientific	STP120
Benchtop autoclave	Astell Scientific	AMB230BT
Brain matrix (mouse coronal 40–75 g)	Electron Microscopy Sciences	Cat# 69080-C
Brush 2.5 mm diameter	Scientific Laboratory Supplies	Cat# BRU2054
Coverslip glass 24 × 50 mm	VWR	Cat# 631-0146
Microscope	Olympus	BX43
Microtome blades, S35 fine, high-profile	VWR	Cat# 720-1998
Microwave	Russell Hobbs	RHM2077
Razor blade	Azpack	Cat# 11904325
Rotary microtome	Leica Biosystems	RM2135
ScanScope slide scanner	Leica Biosystems	AT Turbo
Shandon Plastic Coverplates	Epredia	Cat# 72110017
Shandon Sequenza Immunostaining Center Slide Rack	Epredia	Cat# 73310017
Stainless steel histology mold 37 × 24 × 9 mm	CellPath	Cat# GBB-3714-05A
Stainless steel histology mold 60 × 45 × 15 mm (Supa Mega)	CellPath	Cat# GBC-6014-05A
SuperFrost Plus adhesion slides	Epredia	Cat# J1800AMNZ
Tissue embedding system	Thermo Scientific	HistoStar
Tissue embedding cassette	Kartell	Cat# 0292503
Tweezers/forceps, blunt	Fisher Scientific	Cat# 15576579
Tweezers/forceps, sharp	Fisher Scientific	Cat# 12381369
Warming oven	Memmert	D 06060 model 400

## Materials and equipment

**Table:** Perfusion solution 1

Reagent	Final concentration	Amount
Phosphate buffered saline	1 x	500 mL
Heparin	1 μL/mL	500 μL
Gadovist	2 mM	1 mL
**Total**	**2 mM**	500 mL

***Note:*** Store in the dark.

**Table:** Perfusion solution 2

(For perfusion and fixation)		
Reagent	Final concentration	Amount
Phosphate buffered saline	1x	30 mL
16% paraformaldehyde	4%	10 mL
Gadovist	2 mM	0.8 mL
**Total**	**4%**	**40 mL**

***Note:*** Amount is sufficient for one mouse. Make fresh before use. Dispose of unused solution in accordance with local regulations.

**Table:** Storage solution

Reagent	Final concentration	Amount
Phosphate buffered saline	1 x	499 mL
Sodium azide	0.02%	0.1 *g*
Gadovist	2 mM	1 mL
**Total**	**N/A**	**500 mL**

***Note:*** Store at 4°C. Dispose of unused solution in accordance with local regulations.

**Table:** Phosphate buffered saline (PBS) (pH 7.4)

(For making buffers)		
Reagent	Final concentration	Amount
Phosphate buffered saline	1 x	10 tablets
dH_2_O	N/A	1000 mL
**Total**	**1 x**	**1000 mL**

***Note:*** Mix with a stir bar until fully dissolved. Add HCl and/or NaOH dropwise to adjust pH to 7.4. dH_2_O (distilled water). Store at room temperature (18°C–22°C) for 3 months.

**Table:** 3% H_2_O_2_ in PBS

(For quenching endogenous peroxidases)		
Reagent	Final concentration	Amount
30% H_2_O_2_	3%	30 mL
PBS Buffer	N/A	270 mL
**Total**	**3%**	**300 mL**

***Note:*** Make fresh before use. Store stock hydrogen peroxide at 4°C protected from sunlight. Store working solution at 4°C for up to 3 months. Dispose of unused working solution in accordance with local regulations.

**Table:** Citrate buffer (pH 6.0)

(For antigen retrieval)		
Reagent	Final concentration	Amount
Tri Sodium Citrate	10 mM	2.94 *g*
dH_2_O	N/A	1000 mL
**Total**	**10 mM**	**1000 mL**

***Note:*** Mix with a stir bar until fully dissolved. Add HCl and/or NaOH dropwise to adjust pH to 6.0. Store at room temperature (18°C–22°C) for 3 months or at 4°C for longer.

**Table:** Saline

(For making TBS-T)		
Reagent	Final concentration	Amount
Sodium Chloride	10 x	85 *g*
dH_2_O	N/A	1000 mL
**Total**	**10 x**	**1000 mL**

***Note:*** Mix with a stir bar until fully dissolved. Store at room temperature (18°C–22°C).

**Table:** 0.5 M Tris/HCl stock (pH 7.4)

(For making TBS-T)		
Reagent	Final concentration	Amount
Tris (hydroxymethyl) methylamine	0.5 M	60.5 *g*
4% HCl	0.8%–1.2%	200–300 mL
dH_2_O	N/A	500–800 mL
**Total**	**0.5 M**	**1000 mL**

***Note:*** Mix the Tris with a stir bar in 500 mL dH_2_O until fully dissolved. Add HCl to adjust pH to 7.4 (It requires about 200–300 mL). Add dH_2_O to achieve a total volume of 1000 mL. Add HCl and/or NaOH dropwise to re-adjust pH to 7.4. Store at room temperature (18°C–22°C).

**Table:** Tris buffered saline with Triton X (TBS-T) (pH 7.6)

(Washing solution for immunohistochemistry)		
Reagent	Final concentration	Amount
Saline stock	1 x	90 mL
dH_2_O	N/A	810 mL
0.5 M Tris/HCl stock	0.05 M	100 mL
Triton X	0.05%	500 μL
**Total**	**N/A**	**1000 mL**

***Note:*** Mix with a stir bar for 30 minutes until the Triton-X is fully dissolved. Add HCl and/or NaOH dropwise to adjust to pH 7.6. Store at room temperature (18°C–22°C) for 3 months or at 4°C for longer.

**Table:** DAB working solution

(For visualization)		
Reagent	Final concentration	Amount
Substrate buffer	N/A	980 μL
DAB+ chromogen	1:50	20 μL
**Total**	**1:50**	**1000 μL**

***Note:*** Vortex to mix it. Make fresh just before use and protect from light. Sufficient for 8 slides (120 μL per slide). Store stock components in the dark at 4°C. Unused working solution should be disposed according to local regulations.

**Table:** Harris hematoxylin

(For counter staining)		
Reagent	Final concentration	Amount
Harris Hematoxylin	25%	100 mL
dH_2_O	N/A	300 mL
**Total**	**25%**	**400 mL**

***Note:*** Filter every time before you use it. Hematoxylin working solution should be made fresh after 10 uses. Store stock at room temperature up to 12 months. Store working solution at room temperature (18°C–22°C) for 10 uses.

**Microscope**: We used Olympus BX43 microscope with Zeiss AxioCam MRc camera attachment. Any light microscope should be sufficient to view the slides.

**Slide Scanner**: We used Leica ScanScope AT Turbo at 40x magnification. Any slide scanner achieving this magnification should be sufficient to digitize the slides.

## Step-by-step method details

### Brain dissection

**Timing: 15 min to 1 h per brain depending on the level of experience**

**Figure 2:**
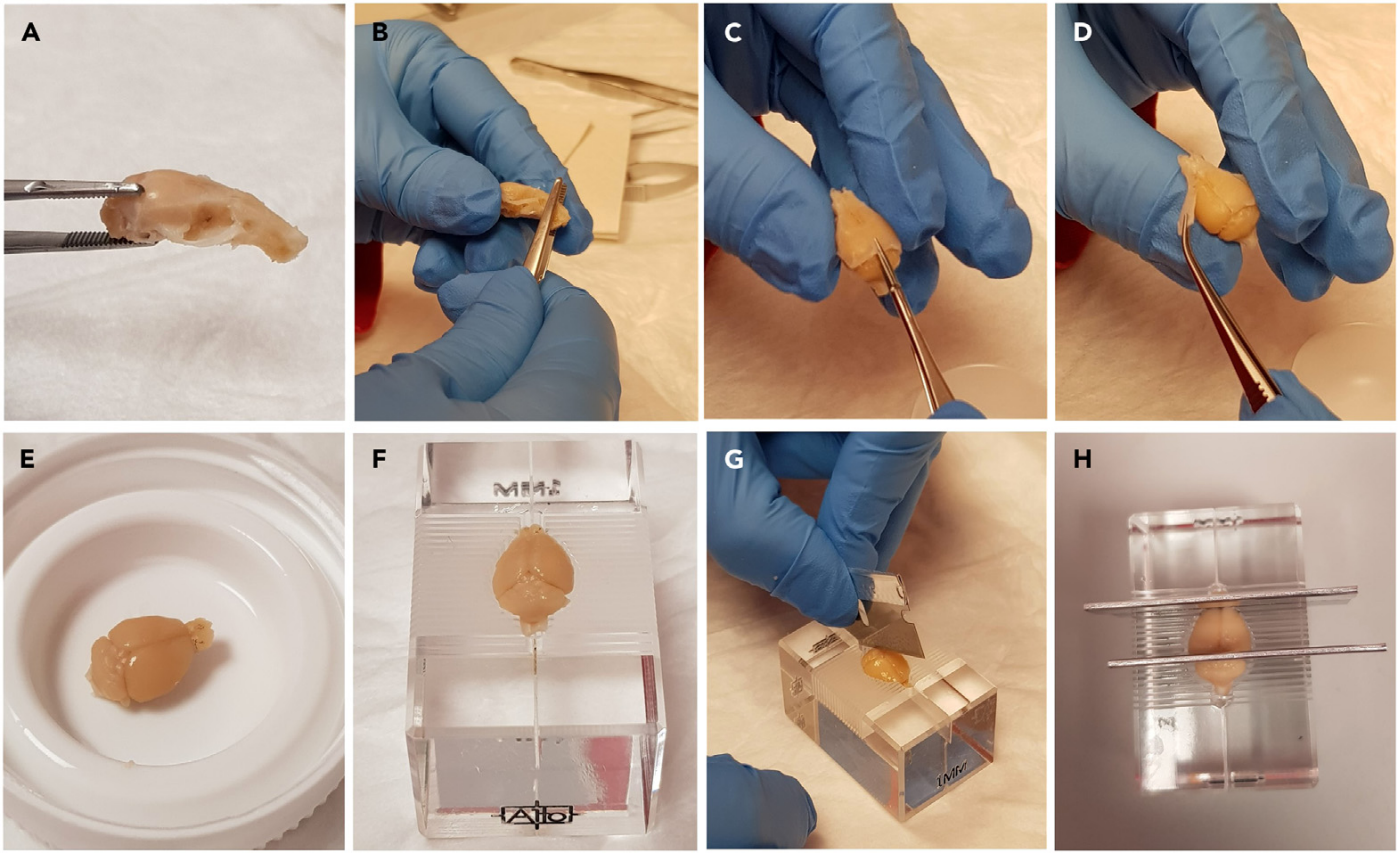
Photos demonstrating the procedure of dissecting the mouse brain (A) Example of mouse brain within the skull. (B‒D) Removing the brain from the skull. (E) Example of brain with the skull removed. (F) Mouse brain within the brain matrix. (G and H) Using a brain matrix to slice the brain. G and H show positioning for sagittal and coronal slicing respectively.

***Note:*** A good step-by-step description is presented by Spijker, 2011.^10^ However, researchers may prefer to use different approaches to remove the mouse brain from the skull depending on the region of interest for histology and on experience.

1. Remove the brain from the skull (Figures 2A‒2E).
  a. Extra care should be taken to avoid damaging the brain with the tweezers or scissors. Doing this process slowly ensures better results.

2. Using a brain matrix (Figure 2F) for adult mouse brains (1 mm between notches), cut the brain with a razor blade at the midline for sagittal sections (Figure 2G) or at notches 4 and 12 for coronal sections (Bregma +3 and ‒5; Figure 2H).

***Note:*** The configuration for coronal sections was done to keep the cerebral cortex in one piece and adhere to the optimized processing protocol (which needs to be adjusted for sample size), removing the olfactory bulbs and the cerebellum. The coronal slab was approximately 8 mm thick after cutting.

***Note:*** While the brain could be divided manually for both coronal and sagittal sections, we recommend using a brain matrix to improve the reproducibility of the cutting plane position and angle.

***Note:*** In case the whole brain is to be assessed with histology in coronal sessions, including olfactory bulbs and cerebellum, we suggest dividing the brain in half and processing the two halves separately. Timing for tissue processing steps may need adjustment depending on the sample size.

3. Store the brain in the storage solution at 4°C until required for tissue processing.

### Processing tissue

**Timing: 20 h**

Processing tissue involves four major steps: fixation, dehydration, clearing and wax infiltration. Formalin is one of the most common fixatives and forms cross-linkages with proteins while preserving morphology and antigenicity.^11^ Dehydration in a series of alcohols is necessary to remove water from the tissue because paraffin wax is immiscible with water. Next, the tissue is cleared by immersing it in a clearing agent, to remove alcohol from the tissue and prepare it for paraffinization. Finally, the tissue is infiltrated with paraffin wax. The advantage of paraffin wax is that it preserves cellular morphology and provides support while sectioning and enables long-term storage.

Sub-optimal processing can result in tissue that is difficult to section^12^ and has poor morphology; therefore, it is vital to optimize the processing protocols for the size and type of tissue. Tissue stored in solution for months to years can pose a particular challenge. The protocol presented here is optimized for adult mouse brain tissue that has been stored in PBS, sodium azide and contrast agent solution after fixation and for up to 27 months before processing. Samples were either one adult brain hemisphere for sagittal sections or an 8 mm-thick slab of the mouse brain for coronal sections, where the olfactory bulbs and cerebellum have been removed (step 2).

4. Rinse the tissue in distilled water twice (Table 2).

**Table 2:** Details of the tissue processing times and temperatures for each step

Step	Reagent	Time	Temperature (°C)
Fixation	Perfusion Solution 1	∼30 min	18‒22
	Perfusion Solution 2	∼30 min	18‒22
	Perfusion Solution 2	12 h	4
Storage for MRI	Storage solution	1–27 months	4
Rinsing	dH_2_O dH_2_O	<1 min	18‒22
		<1 min	18‒22
Dehydration	70% Ethanol	11 h	18‒22
	80% Ethanol	45 min	18‒22
	95% Ethanol-I	45 min	18‒22
	95% Ethanol-II	45 min	18‒22
	100% Ethanol-I	15 min	18‒22
	100% Ethanol-II	15 min	18‒22
	100% Ethanol-III	15 min	18‒22
	100% Ethanol-IV	30 min	18‒22
	100% Ethanol-V	30 min	18‒22
	100% Ethanol-VI	30 min	18‒22
Clearing	CitroClear-I	30 min	18‒22
	CitroClear-II	45 min	18‒22
	CitroClear-III	60 min	18‒22
Infiltration	Paraffin Wax-I	30 min	60
	Paraffin Wax-II	45 min	60
	Paraffin Wax-III	60 min	60

Note: the storage time is based on how long our samples were stored. Distilled water (dH_2_O), minutes (min), hour (h).
5. Place each sample inside its own tissue embedding cassette. Ensure the cassette is deep enough for the size of the tissue.
  a. Label the cassette with the sample identification.
6. Dehydrate the tissue in a series of alcohols at 18°C–22°C. (Ethanol is diluted in distilled water as necessary). Multiple changes of ethanol are required to displace any water.
  a. 70% Ethanol overnight (11 h).
  b. 80% Ethanol 45 min.
  c. 95% Ethanol 45 min, two times.
  d. 100% Ethanol 15 min, three times.
  e. 100% Ethanol 30 min, three times.
7. Clear the tissue in a series of Citroclear at 18°C–22°C. Multiple changes are required to displace all the ethanol.
  a. Citroclear for 30 min.
  b. Citroclear for 45 min.
  c. Citroclear for 60 min.
8. Infiltrate the tissue with paraffin wax through incubation in a series of molten wax at 60°C. Multiple changes are required to displace all the Citroclear.
  a. Paraffin wax for 30 min.
  b. Paraffin wax for 45 min.
  c. Paraffin wax for 60 min.

***Note:*** This tissue processing routine described here is shorter than the recommended routines used for recently collected mouse brains (1 hour per step). When a standard routine is used to process mouse brain tissue after prolonged storage, we found that the blocks are exceedingly difficult to slice, impairing the ability to collect serial sections.

***Note:*** Ethanol, Citroclear and paraffin solutions should be replaced with fresh solutions weekly to maintain quality as cross-contamination may impact the quality of processing.^12^

***Alternatives:*** Histo-Clear II or Xylene clearing agents can be used instead of Citroclear.

### Embedding

**Timing: 2 h**

After processing, samples are embedded in paraffin wax. It is vital that the specimen is carefully orientated into the desired position (usually an orthogonal plane), as this will affect the plane that is cut. It is possible to embed more than one sample in the same block. However, when embedding blocks that contain multiple samples it is important to ensure all samples are level and that enough paraffin is present around each sample. FFPE (Formalin-fixed paraffin embedded) blocks can be stored long term at room temperature (18°C–22°C) because the tissue is preserved within the paraffin. The protocol here describes the method for embedding both single and multiple mouse brains per block.

**Figure 3:**
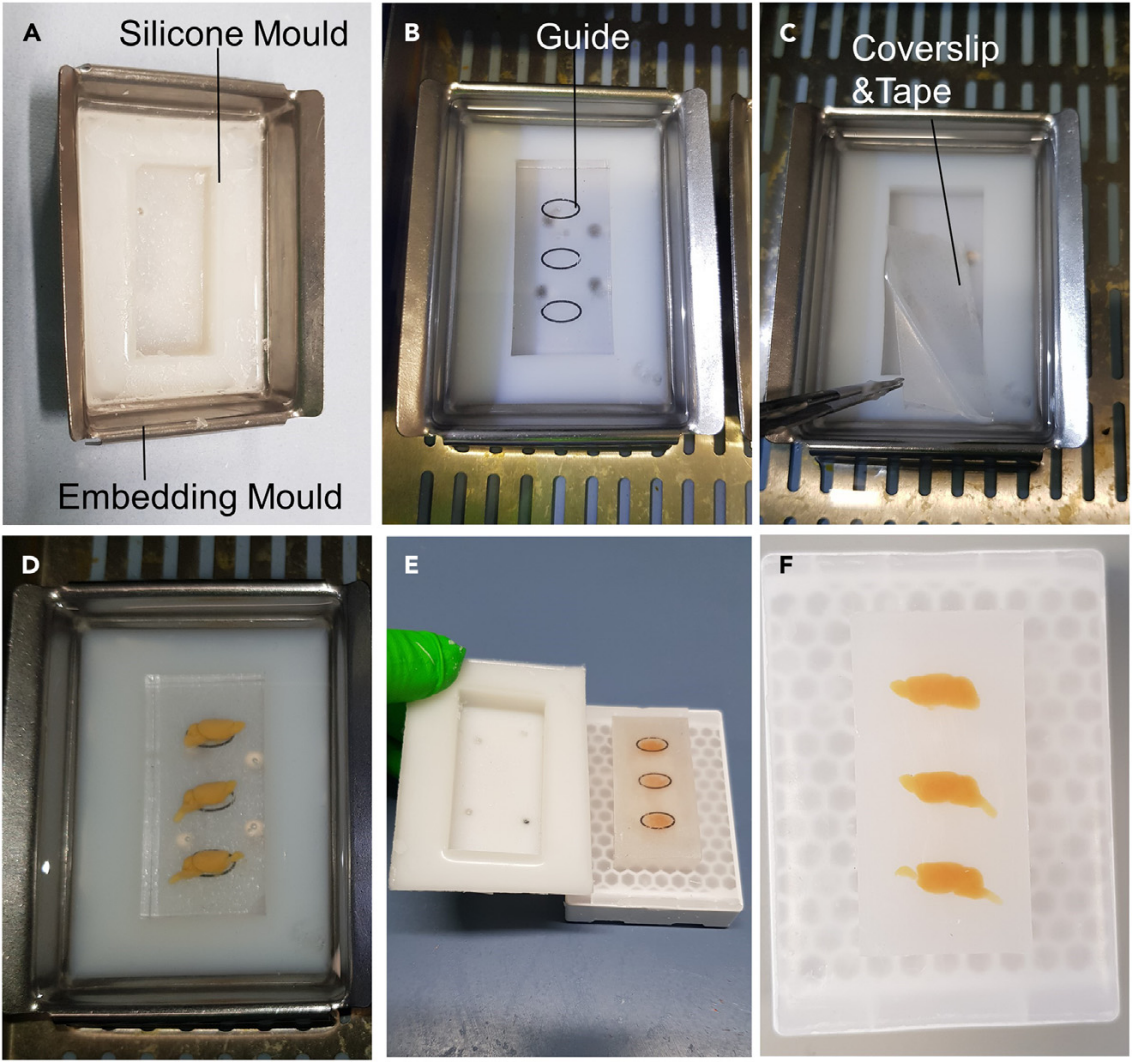
Photos demonstrating the embedding process for multibrain blocks (A) A silicone mold is used to ensure the block size will be suitable for standard 25 mm × 75 mm slides. (B) A printed guide is inserted into the embedding mold, (C) then a glass coverslip with double tape on the top side is placed on top of the printed guide. (D) The guide is used to assist placement of the brains within the molten wax, the coverslip allows to keep all brains on the same plane, and the double tape keeps the brains in place. (E) After the wax has set, the silicone mold, printed guide and coverslip with double tape can be easily removed. (F) Example of a multibrain block with brains positioned for sagittal sections.
12. Place the labeled cassette on top of the mold.
13. Return the mold back to the warm plate and fill with melted paraffin up to the upper edge of the cassette.

***Note:*** Do not overfill the mold as this can interfere with the alignment of the block. Underfilling the mold can cause unstable clamping of the block.

9. Warm the paraffin wax in the embedder 30 min prior to use.
10. Remove the tissue embedding cassettes from the tissue processor and place them inside the cassette bath of the embedder.
11. Prepare the blocks. Tissue can either be arranged to have a single brain per block or multiple samples can be embedded within the same block. Troubleshooting 1.
  a. Prepare single brain blocks.
    i. Pour melted paraffin into a histology mold (e.g., 37 × 24 × 9 mm size mold) until it is partially filled.
    ii. Warm the forceps then remove the specimen from the cassette and transfer it to the histology mold. Orient the specimen into the desired position.
    iii. Transfer the mold onto the cold plate for a few seconds to allow the paraffin to solidify and anchor the brain.
      ***Note:*** Use blunt forceps and gentle pressure to avoid damaging the specimen.
      ***Note:*** Ensure the histology mold is sufficiently deep for the size of the tissue and will allow at least 1 cm of wax between the sample and edge of the block.
  b. Optional**:** Prepare multi brain blocks (Figures 3A–3G).
    i. Insert a silicone mold inside a Supa Mega (60 × 45 × 15 mm) base mold (Figure 3A). We made a silicone mold that measures 50 × 23 × 10 mm internally and 60 × 45 × 14 mm externally.
    ii. Fill the silicone mold with wax.
    iii. Keep the mold filled with warm wax in an oven or the cassette compartment of the embedding station for 1–6 h.
    iv. After allowing the wax to rest, insert a printed guide into the bottom of the mold (Figure 3B).
    v. Insert a coverslip (24 × 50 mm) covered with double tape on top of the printed guide (Figure 3C). The double tape will keep each sample in place and at the right orientation.
    vi. Transfer the brains into the mold according to the guide and record the position of each sample (Figure 3D).
      ***Note:*** Allowing the wax to rest reduces the presence of bubbles in the block. Use tweezers or a spatula to scratch surfaces from time to time and release bubbles that may accumulate. The presence of bubbles in the solidified paraffin wax creates challenges when sectioning and reduces the quality of the histological slides. While bubbles are not common in small size paraffin blocks, they are more common in bigger blocks.

14. Transfer the mold to the cold plate and allow the wax to fully solidify, ideally for up to 30 min. Then separate the mold from the cassette.

***Note:*** For multi brain blocks, the custom silicone mold, and printed guide can be easily separated from the block after removing it from the mold. Remove the coverslip/double tape carefully.

15. Assess the quality of the block and tissue. Troubleshooting 1.
  a. The wax should be free from cracks or air bubbles.
  b. The sample should be orientated correctly and should not be touching the edge of the block.
  c. There should be no smell of Citroclear. If present, this suggests that either the Citroclear was not completely removed during processing, or the tissue may be under-processed. Troubleshooting 2.

**Pause point:** Blocks can be stored at 18°C–27°C long term until needed for sectioning.

### Sectioning

**Timing: 1–3 h per brain block**

Sectioning can introduce artefacts that will impact downstream registration and analysis. Therefore, it is vital care is taken to reduce this. We found that soaking the blocks in Mollifex and ice-cold water prior to sectioning and decreasing slice thickness to 4–6 μm improved the quality of the slices.

16. Fill the water bath with distilled water and heat to 42°C.

***Note:*** Despite the general recommendation to use water at 5°C–10°C below the melting point of paraffin,^13^ we found that it led to overexpansion and breakage of the mouse brain tissue. Using a lower temperature of 37°C–42°C allowed wrinkles to be removed without damaging the tissue due to overexpansion and loss of morphological structure.

17. Chill the block on the surface of ice for <5 min.

***Note:*** Do not place the blocks in the freezer as this will damage them.

**CRITICAL:** Label the slides with the block ID and serial section number.

18. Remove any excess wax from the outside of the cassette before clamping the block.
19. Set the paraffin block into the microtome. Ensure the block is parallel to the blade and adjust the angle of the blade. 2–5 degrees is the recommended clearance angle for paraffin blocks.^13^ It is important that the tissue is sectioned perpendicularly.
20. Trim the block at 10–30 μm until a full faced section is achieved.

***Note:*** Sections can be viewed under the microscope to view the anatomy and check if the angle of the block requires adjusting. Troubleshooting 3.

**Figure 4:**
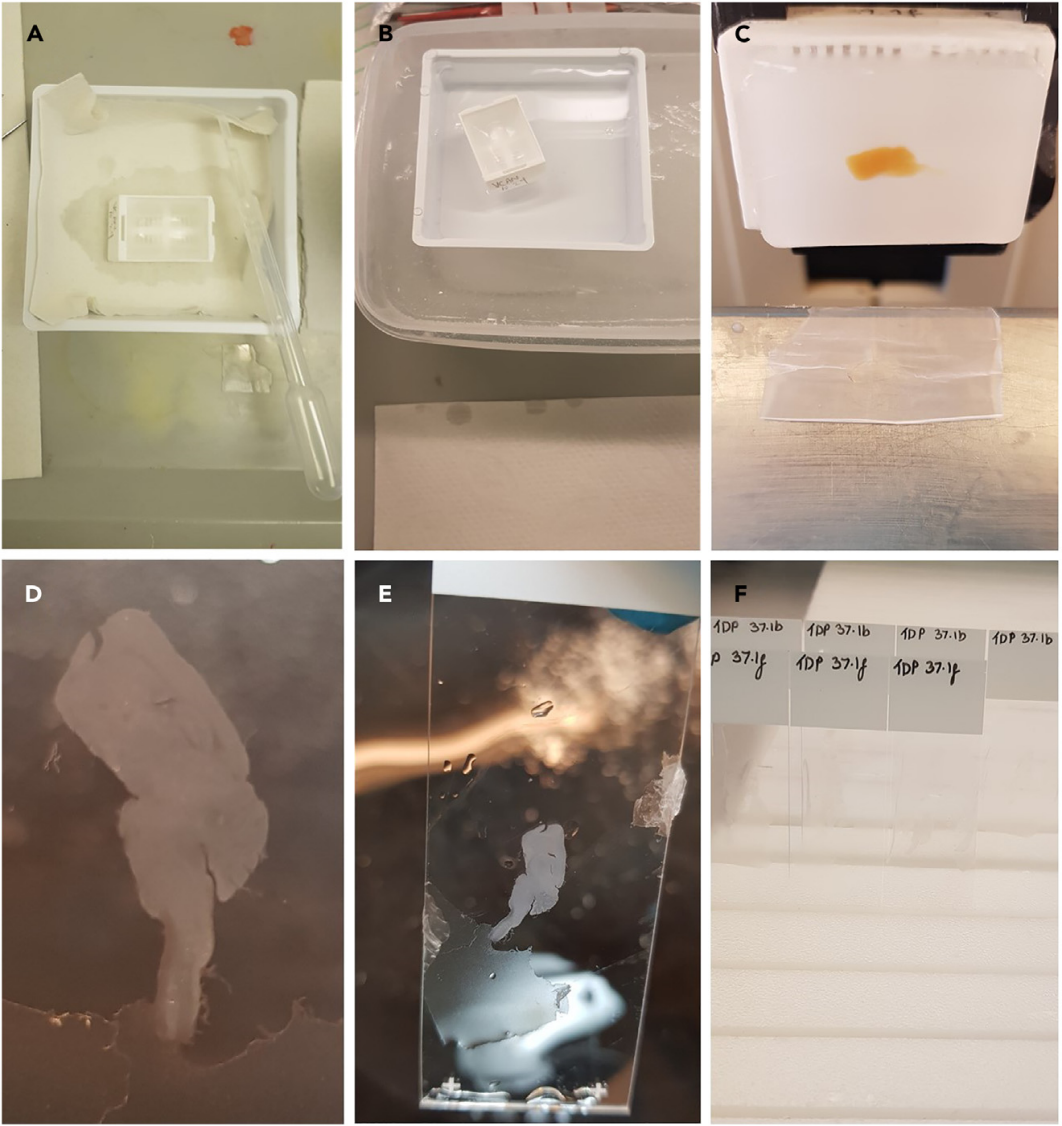
Microtome workflow (A) Soak the block in Mollifex followed by (B) ice water. (C) Cut sections. (D) The section is floated on a warm water bath until smooth and then (E) placed on a glass slide. (F) Sections are allowed to dry upright at 18°C–22°C.

***Note:*** Mollifex should be used in a fume hood. Users should wear gloves, protective clothing and safety glasses.

21. After the block is trimmed, place the block in Mollifex reagent for at least 30 min prior to sectioning. This step will help soften hard or brittle tissue (Figure 4A).

22. Gently wipe away the Mollifex and then chill the block briefly on the surface of melting ice for up to 5 min (Figure 4B).

***Note:*** Avoid prolonged soaking in ice water. This can cause the tissue to swell and may damage the tissue morphology.

23. Section the block slowly at 4–6 μm with a uniform rotation (Figure 4C). Pick up each section using either a small brush or small tweezers. Troubleshooting 4**.**
  a. Discard the first two sections of a ribbon of consecutive sections. These sections are usually thicker than desired due to expansion of the cold block. We took up to 20 serial sections.

***Note:*** Gentle warm breath on the section can prevent sections rolling up and reduce static electricity.

***Note:*** If the tissue shows signs of becoming dry at the edge of the tissue, it is necessary to repeat the chilling step before more sections can be taken.

**CRITICAL:** Avoid touching the blade with the brush or tweezers as this may cause nicks in the blade resulting in scoring on the tissue.

24. Transfer the section onto the water bath and allow the section to float on the surface until there are no wrinkles (<15 s) (Figure 4D). Do not float the section on the water for longer as expansion may distort the tissue.^13^ Avoid trapping air bubbles underneath the section on the surface of the water bath.
25. Collect the section onto a charged glass slide (Figure 4E). Be careful to avoid trapping air bubbles underneath the tissue when transferring them onto the glass slide.
26. Dry in an upright position overnight (>18 h) at room temperature (18°C–22°C) to allow excess water to drain (Figure 4F). Then store in a slide box.
27. Assess the quality of the sections.
  a. Sections should be free from wrinkles, folds, scratches and artefacts. Troubleshooting 3 and 4.
  b. The brain should remain in the anatomically correct position.
  c. The brain should not be positioned close to the edge of the glass slide.
  d. There should be no air bubbles trapped underneath the section.
  e. Sections should be free from any debris or contamination.

***Note:*** Minimize handling of slides to avoid contaminating them with squamous cells or oils. Only use clean slides and do not touch glass slides or the water bath without gloves. We suggest using charged slides to avoid adding a coating step.

**Pause point:** The unstained slides can be stored at in the dark at 18°C–27°C for up to 1 year. Avoid prolonged exposure to direct sunlight. However, the literature has found long term storage of pre-cut sections can result in reduced staining intensity of some markers.^14^

### Immunohistochemistry

**Timing: 5–6 h**

This section describes a standard immunohistochemistry protocol optimized for the antibodies and tissue used in this study. Immunohistochemistry allows us to detect the location of proteins and antigens in tissue using specific antibodies (Table 3). The main steps are tissue preparation, antigen retrieval, blocking, antigen binding, visualization and counterstaining. This protocol describes the indirect antibody binding method which allows for amplification of the signal. The antigens are visualized using the chromogen HRP-DAB (3, 3-diaminobenzidine). Hematoxylin is a common counterstain, which stains for basophilic structures including cell nuclei, and is used in this protocol.

**Table 3:** Detailed staining conditions for each primary antibody used for mouse FFPE sections

Antibody	Target	Host	Clonality	Antigen retrieval	Working dilution and incubation time
Anti-Brevican (BCAN)	Extracellular matrix, perineuronal nets	Rabbit	Monoclonal	Autoclave, Citrate pH 6	1:2,000, 1 h, 18°C–22°C
Anti-CD68	Macrophages and activated microglia	Rabbit	Polyclonal	Autoclave, Citrate pH 6	1:400, 1 h, 18°C–22°C
Anti-Ferritin FTH1	Iron storage	Rabbit	Monoclonal	Autoclave, Citrate pH 6	1:500, 1 h, 18°C–22°C
Anti-Myelin PLP	Myelin	Rabbit	Polyclonal	Microwave, Citrate pH 6	1:500, 1 h, 18°C–22°C
Anti-NeuN	Mature neurons (nuclei)	Rabbit	Monoclonal	Autoclave, Citrate pH 6	1:3,000, 1 h, 18°C–22°C
Anti-Neurofilament 200 (NF)	Neurofilament heavy polypeptide	Rabbit	Polyclonal	Autoclave, Citrate pH 6	1:2,000, 1 h, 18°C–22°C

Hour (h).

**Figure 5:**
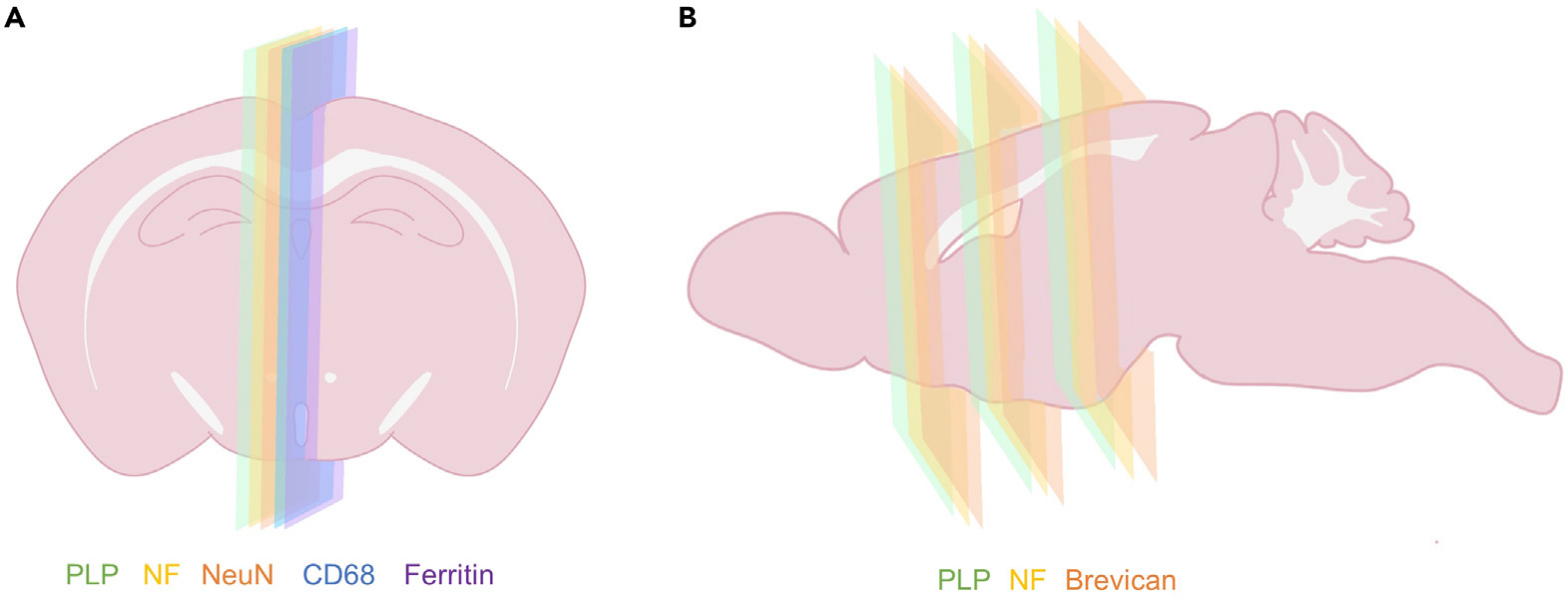
Example staining strategy (A) For a single region of interest, we acquired neighboring sections with different stains, such as PLP, Neurofilament (NF), NeuN, CD68 and Ferritin close to the midline. (B) For multiple regions of interest, we acquired three different stains, PLP, NF, Brevican, per region of interest.

In this protocol, we stained for multiple antibodies on neighboring slides for each sample (Figure 5). To enable histology-to-histology alignment (registration), it is vital that each slide with a different stain neighbor the next within the region of interest. Ideally, the sections should be consecutive; however, it may be necessary to skip a few sections in order to obtain the best quality section. In this case, the gap should still be small enough to allow proper alignment of different histological slides.

28. Bake the slides for 25 min at 60°C in a warming oven.
29. Deparaffinize and rehydrate the sections using sequential immersion. This should be performed under a fume hood or at a table with downdraft ventilation.
  a. 5 min in xylene three times.
  b. 100% Industrial denatured alcohol (IDA) twice. Submerge and lift 10‒20 times.
  c. 90% IDA in distilled water. Submerge and lift 10‒20 times.
  d. 70% IDA in distilled water. Submerge and lift 10‒20 times.
  e. Soak in tap water for 2 min.

**CRITICAL:** Xylene is toxic and flammable. Inhaling xylene vapor can cause respiratory irritation and may damage organs through prolonged exposure. Always use it inside a fume hood or at a ventilated staining table. Wear protective gloves, protective clothing and safety glasses. Alternative reagents that are less toxic are available.

***Alternatives:*** Histo-Clear II or Citroclear clearing agents can be used as a less toxic alternative instead of Xylene.

**CRITICAL:** Xylene and alcohol solutions can be reused but should be changed weekly. Cross contamination of solvents, such as water in xylene, may lead to artefacts. Dispose as hazardous waste in accordance with local regulations.

30. Block endogenous peroxidases by submerging the slides in 3% H_2_O_2_ in PBS in a plastic dish. Make fresh before use. Place on an orbital shaker at 18°C–22°C for 30 min.
  a. After that, immerse the slides in water to rinse for 1 min.

***Note:*** It is necessary to block endogenous peroxidases when using HRP conjugated antibodies as non-specific or high background staining may occur due to endogenous peroxidase activity.

**Figure 6:**
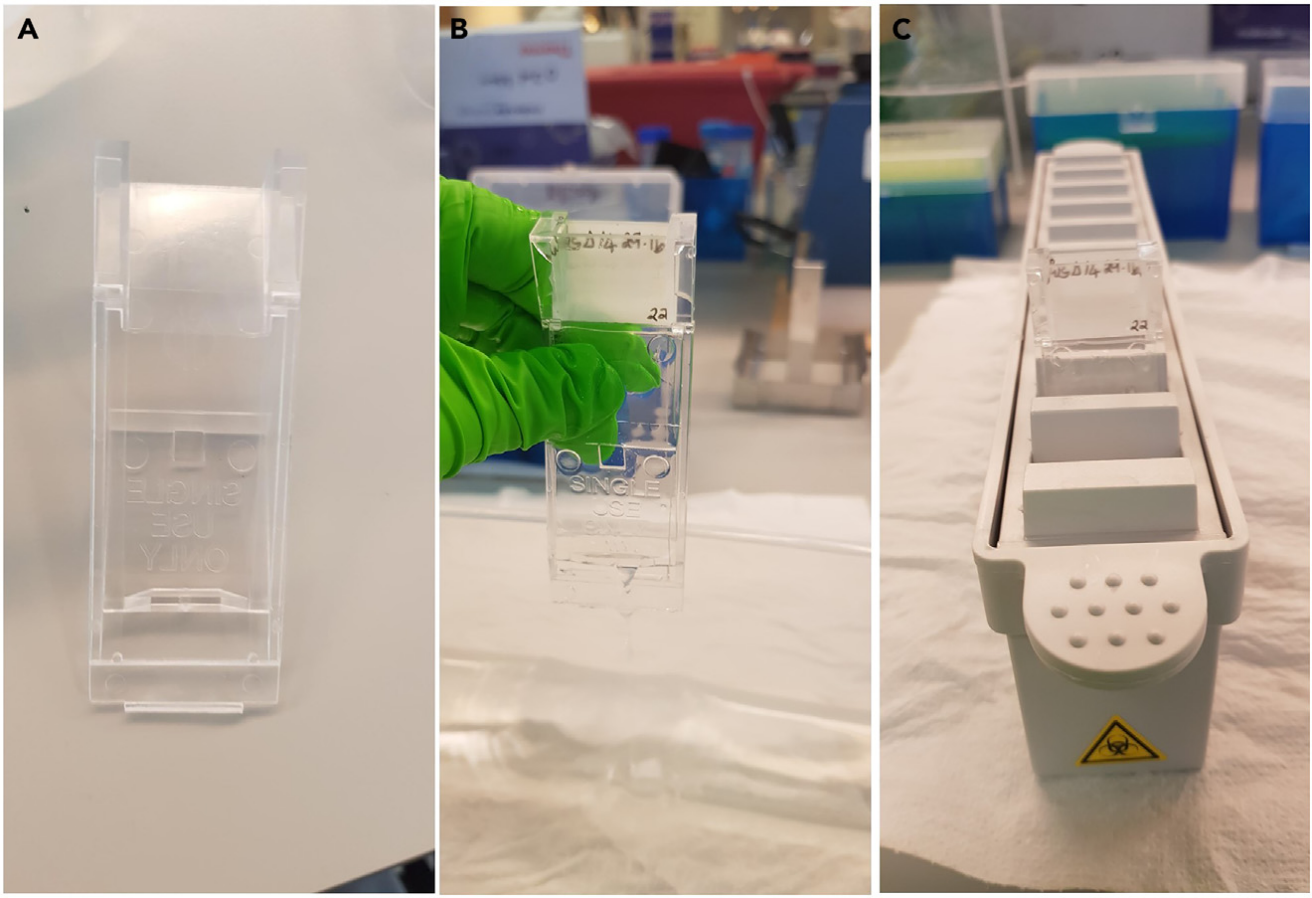
Photos of coverplates and slide rack used during immunohistochemistry (A) Coverplate. (B) Immerse the coverplate into water ensuring there are no bubbles on the surface of the coverplate then place the slide on top of the coverplate with the tissue facing the coverplate. Lift the coverplate/slide out of the water while squeezing slightly to prevent water leaking then insert the coverplate with slide into the rack (C).

***Note:*** Do not use any coverplates that have scratches on the surface as this can affect the quality of the staining. Avoid trapping air bubbles between the slide and coverplate because this will cause staining artefacts.

31. Perform heat-mediated antigen retrieval if required using either a microwave or autoclave. This step is dependent on which antibody is being used (Table 3). Troubleshooting 5.
  a. Microwave
    i. Immerse slides in citrate buffer (pH 6) in a plastic staining dish. Do not seal the dish to allow for evaporation.
    ii. Heat in a microwave for 3 min on high power (800 W).
    iii. Allow it to cool for 5 min at room temperature (18°C–22°C).
    iv. Reheat in a microwave for 1 min.
    v. Allow it to cool for 5 min at room temperature (18°C–22°C).
    vi. Reheat in a microwave for 1 min.
    vii. Slowly cool by gently running water into the staining dish until cool. Do not submerge slides straight into cold water.
      ***Note:*** Optimal heating time may vary depending upon the wattage of the microwave. Aim to achieve a constant temperature of 95°C for 10–20 minutes.
      **CRITICAL:** During antigen retrieval the solution may boil over and evaporate. Do not allow the level of the solution to fall below the level of the slides as the tissue may dry out. Add more solution if needed.
  b. OR Autoclave
    i. Immerse slides in citrate buffer (pH 6) in a plastic staining dish and place these on a metal tray. Do not seal the dish to allow for evaporation.
    ii. Run an antigen retrieval program (121°C, 15 psi, 10 min).
    iii. Slowly cool by gently running water into the staining dish until cool. Do not submerge slides straight into cold water.
      ***Alternatives:*** Heat-mediated epitope retrieval can also be performed using either a pressure cooker or steamer.
      ***Note:*** Antigen retrieval is often required because formalin fixation creates crosslinks with proteins which may mask the antigen of interest.^15^
      ***Note:*** Some antibodies may be temperature sensitive, so it is recommended that the optimal temperature, buffer, pH, duration, and heating device be determined for each individual antibody for heat-mediated heat retrieval. We recommend making gradual changes to temperature, time and pH if the initial set up does not work.

32. Mount the slides into the staining coverplates (Figure 6). These coverplates use capillary action to keep slides hydrated and reduce the amount of antibody required per slide.
  a. Mount the glass slide to the coverplates under water.
  b. Remove the coverplate from the water while gently squeezing it.
  c. Slot the coverplate with slide into the Shandon Sequenza Rack until it clicks into position.

***Alternatives:*** This protocol uses upright Epredia Shandon Sequenza Staining Coverplates and Racks, but flat staining trays can also be used. If using a flat staining tray, it is important to ensure that the tissue does not dry out. Keep a moist paper towel in the bottom of the tray and cover the tray to create a humid environment. Larger volumes of antibodies and DAB may be required to ensure the tissue is fully covered.

33. Wash with TBS-T for 5 min twice.

**CRITICAL:** During this first wash step it is important to check the speed at which the slides drain. If the coverplate empties immediately, remount the slide to the coverplate or mount it to a new coverplate.

34. Incubate the slides with Protein Block solution at 18°C–22°C for 1 h.
  a. We used 120 μL per slide.
  b. Do not wash the blocking solution off after.

***Note:*** Protein blocking can prevent non-specific binding of antibodies to Fc (fragment crystallizable) receptors or tissue.

***Alternatives:*** Alternatives such as fetal bovine serum or a serum matching the species of the secondary antibody can be used to block non-specific binding of antibodies to the tissue.

35. Make the primary antibody working solution by diluting the primary antibody in antibody diluent reagent. We used the dilutions described in Table 3. The working solution should be made fresh before use and mixed well. When optimizing, a range of dilutions should be tested. The optimal dilution is the one that results in specific staining with low background.^15^

***Alternatives:*** The primary antibody can also be diluted in either TBS-T or PBS.

36. Incubate the slides with the primary antibody for 1 h at 18°C–22°C. We advise including both positive (tissue known to contain the target antigen) and negative (no primary antibody) control slides to monitor experimental quality and reproducibility. Troubleshooting 6.
  a. We used 120 μL per slide.
37. Wash with TBS-T for 5 min twice.
38. Incubate the slides with the secondary antibody for 1 h at 18°C–22°C. The secondary antibody we used in this protocol does not require dilution as it is ready-to-use.
  a. We used 100 μL per slide.
39. Wash with TBS-T for 5 min twice.
40. Incubate slides with DAB working solution for 5 min at 18°C–22°C. It is essential to use the same incubation time for every batch to prevent variation between batches.
  a. Create a 1:50 dilution of DAB+ chromogen in substrate buffer (Liquid DAB+ Substrate Chromogen System Kit). Prepare solution just before use.
  b. Add 120 μL of solution per slide.

**CRITICAL:** Do not store DAB working solution or its components in direct light.

**CRITICAL:** DAB is suspected of causing genetic defects and may cause cancer. Wear protective gloves, protective clothing and safety glasses.

41. Quench the DAB with TBS-T for 5 min.
42. Transfer the slides from the coverplates to a staining rack.
  a. Transfer the slides underwater to prevent tissue drying.
43. Counterstain the slides in hematoxylin for 1–5 min. Filter the hematoxylin before using.
44. “Blue” the slides under running water that is weakly alkaline for 3–5 min. This helps to convert the soluble red color of hematoxylin to insoluble blue.

***Note:*** Sections can be quickly viewed under the microscope after counterstaining to check the level of counterstain is sufficient to visualize cellular anatomy. If the hematoxylin is too pale the sections can be returned to the hematoxylin solution.

45. Dehydrate and clear the slides in sequential staining troughs.
  a. 70% IDA in distilled water. Submerge and lift 10‒20 times.
  b. 90% IDA in distilled water. Submerge and lift 10‒20 times.
  c. 100% IDA twice. Submerge and lift 10‒20 times in each of the two troughs.
  d. Xylene, three times. Submerge and lift 10‒20 times in each of the three troughs.

**CRITICAL:** Do not use the same 100% ethanol and xylene that was used to deparaffinize and rehydrate the sections in step 29 instead use separate staining troughs. This step should be performed under a fume hood or at a table with downdraft ventilation.

46. Mount the slides onto glass coverslips with DPX. This should be performed in a fume hood.
  a. Lay a coverslip down and add 1–3 drops of DPX onto the surface.
  b. Remove the slide from the xylene and tap off excess xylene from the slide.
  c. Place the slide facedown onto the coverslip. Ensure slide and coverslip are well aligned.
  d. Remove any air bubbles by gently applying pressure to the coverslip.

**CRITICAL:** DPX contains xylene and is flammable and toxic. Always use it inside a fume hood or at a ventilated staining table. Wear protective gloves, protective clothing and safety glasses.

47. Allow slides to dry in the fume hood overnight (>12 h) at room temperature (18°C–22°C).
48. Assess the quality of the stained sections.
  a. Staining is specific and without high background. Staining should look even. Troubleshooting 6.
  b. No artefacts are present. For example, no negative areas caused by air bubbles trapped in the coverplate are observed on the stained slide.
  c. Tissue is not damaged and has not lifted off the slide. Troubleshooting 5.

**Pause point:** Stained slides can be stored at room temperature (18°C–27°C) until needed for digitization. Slides can be stored for years.

### Registration

**Timing: 30 min to 1 h**

This section describes how to align the histology sections to the corresponding MRI data in the same sample. Each digitized histology slide was registered to 3D T2-weighted structural MRI volumetric data using tensor image registration library^16^ (TIRL; v3). The parameters for the T2-weighted data acquisition details are described in (Table 1).

**Figure 7:**
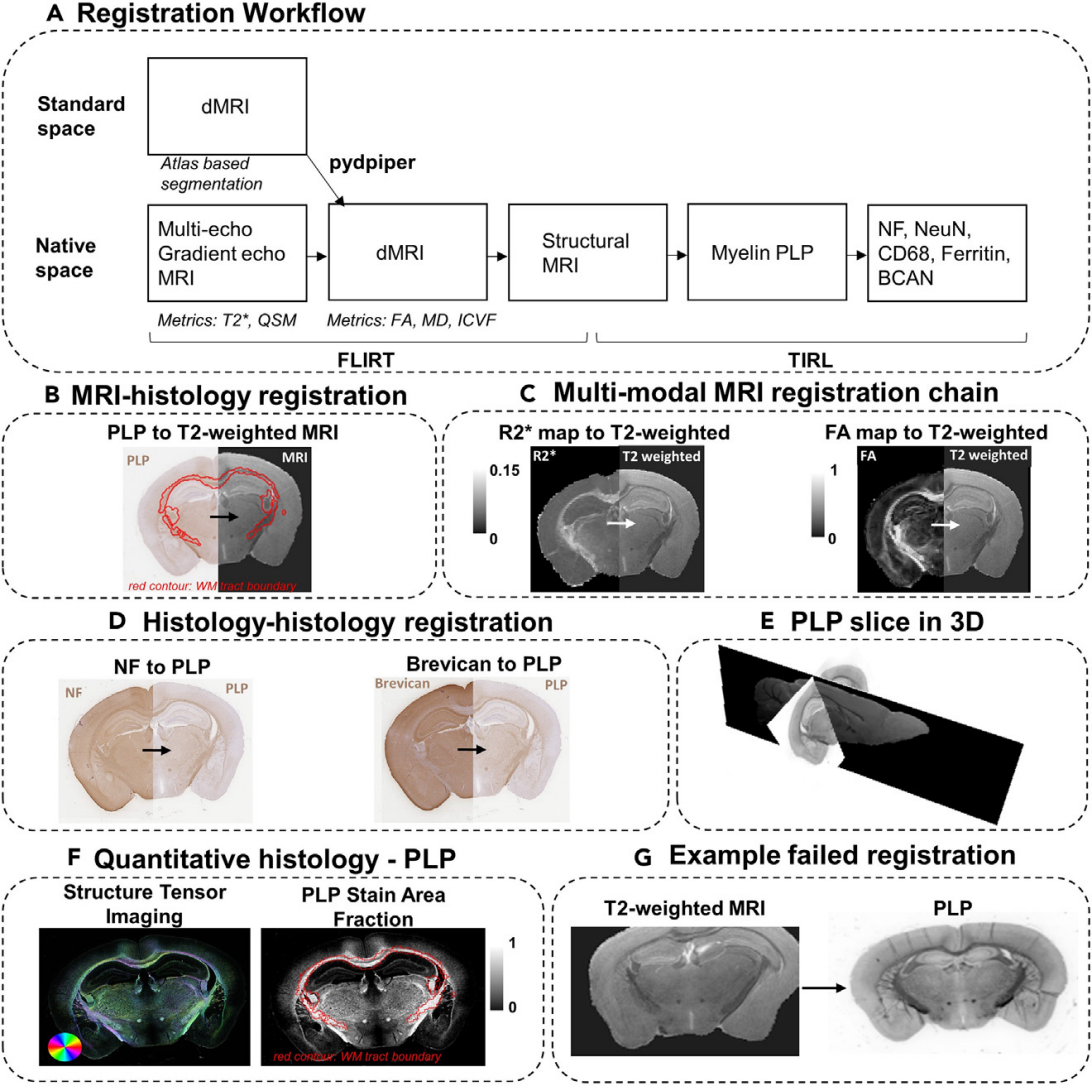
MRI-histology registration chain and examples of outcomes Framework for histology-MRI registration and extraction of quantitative histology metrics. (A) Multi-modal MRI to multi-stain histology registration workflow. Pydpiper is used for the standard-space registration and atlas-based segmentation of the dMRI data. FSL-FLIRT is used to co-register the multi-modal MRI data. TIRL is used for the MRI-histology and histology-histology registration. (B) Example MRI-histology (PLP) registration, red contour line represents the white matter boundary. (C) Example Multi-modal MRI registration result. R2∗ is registered to T2-weighted MRI, and FA (Fractional anisotropy) is registered to T2-weighted MRI. (Note FA was calculated using the diffusion tensor model). (D) Example histology-histology registration result. Neurofilament (NF) is registered to the PLP section, and Brevican is registered to the PLP section for the same sample. (E) PLP slice (coronal) displayed in 3D MRI space (represented by sagittal slice). (F) Quantitative histology metrics obtained from PLP: Structure tensor imaging and Stain Area Fraction map. The color wheel represents the fiber orientation. Red contour represents the white matter tract boundary. The white matter mask was manually segmented in standard diffusion-weighted MRI space with a visual aid from an atlas-based segmentation output^16^ and a tract skeleton^23^ (G) Example of a failed registration of an overprocessed sample which has shrinkage and folds.

Slides stained with the rabbit anti-myelin proteolipid protein (PLP) antibody (see key resources table) are directly registered to the T2-weighted MRI volume for the same sample. Myelin PLP data allows for good quality MRI-histology registration due to its excellent gray-white matter contrast. This aids the registration algorithm, which relies on edges present in the two images. The myelin PLP slides were chosen as the reference histology data for histology-to-histology registration of the neighboring slides. For example, we register the PLP histology slide to MRI, then we register the neighboring neurofilament slide to the PLP slide (Figure 7). Each 2D slide is registered individually. We subsequently extract quantitative histological metrics (stain area fraction maps^17^ and structure tensor maps^18^) from our digitized slides (Figure 7F).

49. Digitize the stained slides at 40x using a digital slide scanner (resolution: 0.25 μm/pixel).
50. Process the multi-modal MRI data (Figure 7A).
  a. Correct the T2-weighted data for 3D Gibbs-ringing.^19^^,^^20^
  b. Extract the brain (remove the skull) from images. This can be done using an atlas-based segmentation^21^ obtained following a non-linear registration to a cohort-specific standard space, using the Pydpiper tool.^22^
51. Co-register the multi-modal MRI data (T2-weighted, T2∗-weighted and dMRI) using a rigid registration tool such as FLIRT^23^^,^^24^ (FMRIB Linear Image Registration Tool; Figures 7A and 7C).
52. Run the histology-to-MRI registration pipeline using TIRL. We recommend registering PLP histology to T2-weighted MRI due to their good white and gray matter contrast (Figure 7B).
  a. Run TIRL after inputting the following parameters into the configuration file (slice_to_volume.json):
    i. The locations of the MRI and histology files.
    ii. The locations of the log files.
    iii. The image resolutions.
    iv. The slicing plane and direction (coronal, axial or sagittal).
    v. Approximate location of the slab (center coordinates), as determined from FSLeyes.^9^
    vi. Input and output directories.
    vii. Optional**:** Specify a mask image for either the MRI or histology data. This can be, for example, a brain mask, or a manually segmented mask. The registration can be performed for regions exclusively inside or outside the mask, depending on requirements. For instance, we might only want to conduct the registration for the region inside the brain mask, or we might want to exclude the region inside the manually segmented mask.

***Note:*** An example configuration file for histology-to-MRI registration can be found here https://git.fmrib.ox.ac.uk/ihuszar/tirl/-/blob/v3/scripts/mouse/slice_to_volume.json?ref_type=heads.

***Note:*** Steps i-vi present the default histology-to-MRI registration protocol. Depending on requirements, this can be further optimized. Consult the comments next to each parameter in the configuration file if you need to improve the registration outcome.

***Note:*** The MRI-histology registration pipeline includes rigid, affine, in-plane and free-form deformations. It can apply linear and non-linear transformations to account for tissue deformations and for variable angle of the slicing plane.

53. Evaluate the quality of registration using Fiji^8^ (Figure 7).
  a. Images are well aligned and anatomically correct. The edge of the tissue and gray/white matter boundaries match between MRI and histology.
  b. The images are not distorted as demonstrated in Figure 7G. This faulty registration outcome due to too many folds and breaks present in the sample.
54. Register the neighboring histology slides acquired using different stains to the PLP slide (Figure 7D).
  a. Run TIRL adjusting the following parameters in the configuration file (histology_to_histology.yml):
    i. The locations of the histology files.
    ii. The locations of the log files.
    iii. The image resolutions.
    iv. Optional**:** Specify a mask image for either file.

***Note:*** An example configuration file for histology-to-histology registration can be found here: https://git.fmrib.ox.ac.uk/ihuszar/tirlscripts/-/tree/master/tirlscripts/oxford/mouse/histology_to_histology.yml.

***Note:*** The histology-to-histology registration includes rigid, followed by affine and non-linear deformations.

55. Assess the quality of the histology-to-histology registration outcome. Check that:
  a. Images are well aligned and appear anatomically correct. The edge of the tissue and gray/white matter boundaries match between neighboring slides stained for different histological markers.
  b. The images are not distorted.

***Note:*** If the input file for the histological image is in svs format, it is recommended that a lower resolution image is used for registration to reduce computational time. To do this, you need to edit the “slice”: “pages” parameter in slice_to_volume.json. We use “pages”: [2], which will specify a lower-resolution image from the .svs resolution pyramid. This corresponds to a resolution of 4 μm/pixel.

### Quantitative assessment: SAF maps

**Timing: 2 h**

SAF (stained area fraction) maps allow for quantitative measurement of immunohistochemistry stains by extracting the number of DAB-positive pixels within a given area.^17^ SAF maps are extracted using the ihcpy pipeline. Scripts can be found at https://git.fmrib.ox.ac.uk/spet4877/ihcpy/-/tree/main/. This pipeline allows us to analyze MRI and histology data voxelwise and can be generalized to multiple immunohistochemical stains. The pipeline involves: using color deconvolution to separate DAB and hematoxylin stains, segmenting DAB specific staining from non-specific staining, and calculation of the SAF map.^17^ Figure 7F shows an example SAF map with the white matter boundary highlighted.

Here we present the default pipeline. An artefact pipeline is also available for images that show a strong striping effect (produced when scanning the slide) or staining gradient.

56. Run the optimization pipeline (iterate_alphas.sh) and adjust the following parameters. The threshold should be optimized for each different stain.
  a. The location of the histology image.
  b. The output directory.
  c. The starting alpha, alpha increments and end alpha for the range of alphas to iterate.
  d. Segmentation dimension.
  e. Patch size.
  f. The location of logfiles.
57. Examine the segmentation images for each threshold to determine the best threshold for that stain.
  a. The best threshold is one without noise that accurately extracts the stain.
58. Run the default pipeline (default_pipeline.sh) and adjust the following parameters:
  a. The location of the histology image.
  b. The output directory.
  c. Weighting for segmentation (threshold).
  d. Segmentation dimension.
  e. Patch size for high and low resolution SAF.
  f. The location of logfiles.

## Expected outcomes

This protocol describes tissue processing, embedding, sectioning, staining and registration methods for adult mouse brains. Although our lab used DAB immunohistochemistry, histology stains such as cresyl violet (Nissl) can be used instead.

**Figure 8:**
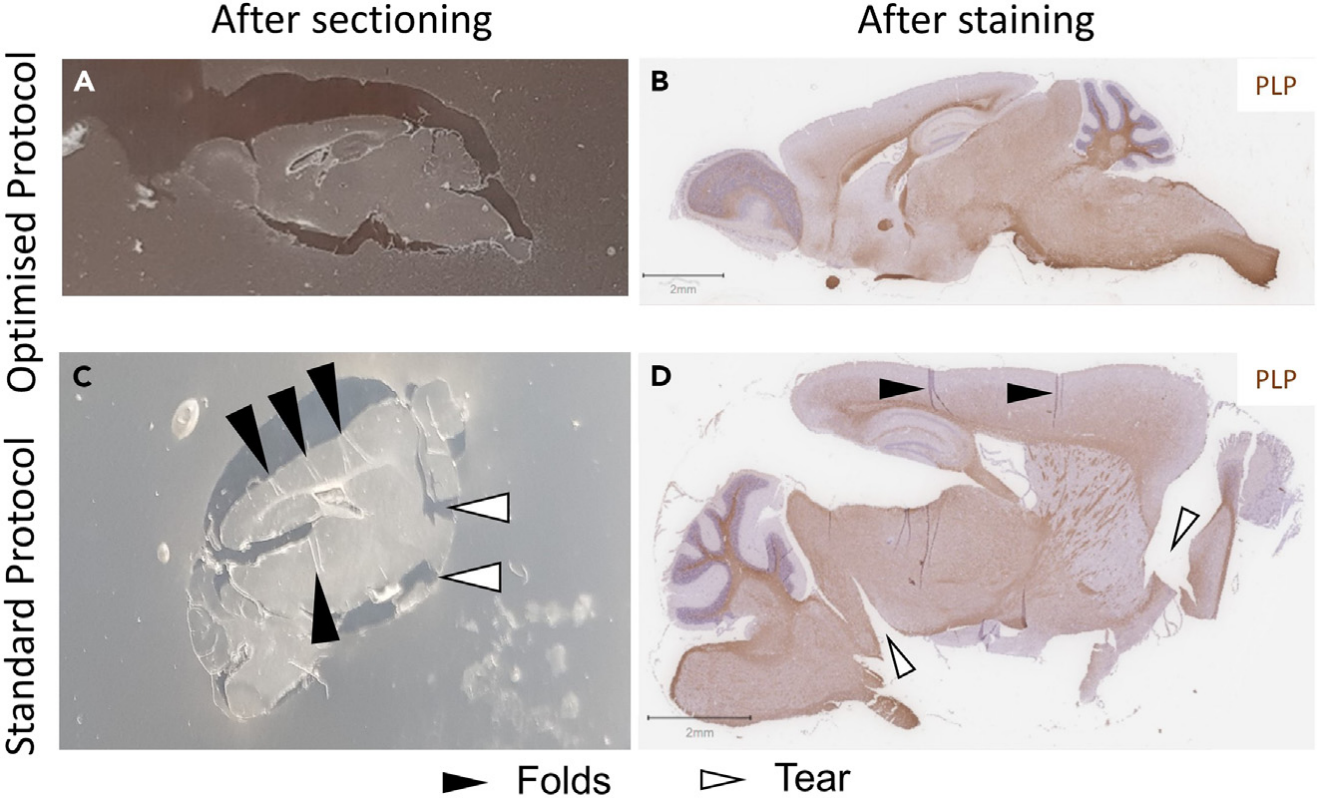
Representative images comparing outcomes of the optimized and standard protocols (A and B) Representative images of the optimized protocol after sectioning (A) and after PLP staining with hematoxylin counterstaining (B). (C and D) Representative images of the standard protocol after sectioning (C) and after PLP staining with hematoxylin counterstaining (D). The representative images were sectioned and stained by the same operator. Folds (black arrowhead). Tears (white arrowhead). Scale bar is 2 mm.

**Figure 9:**
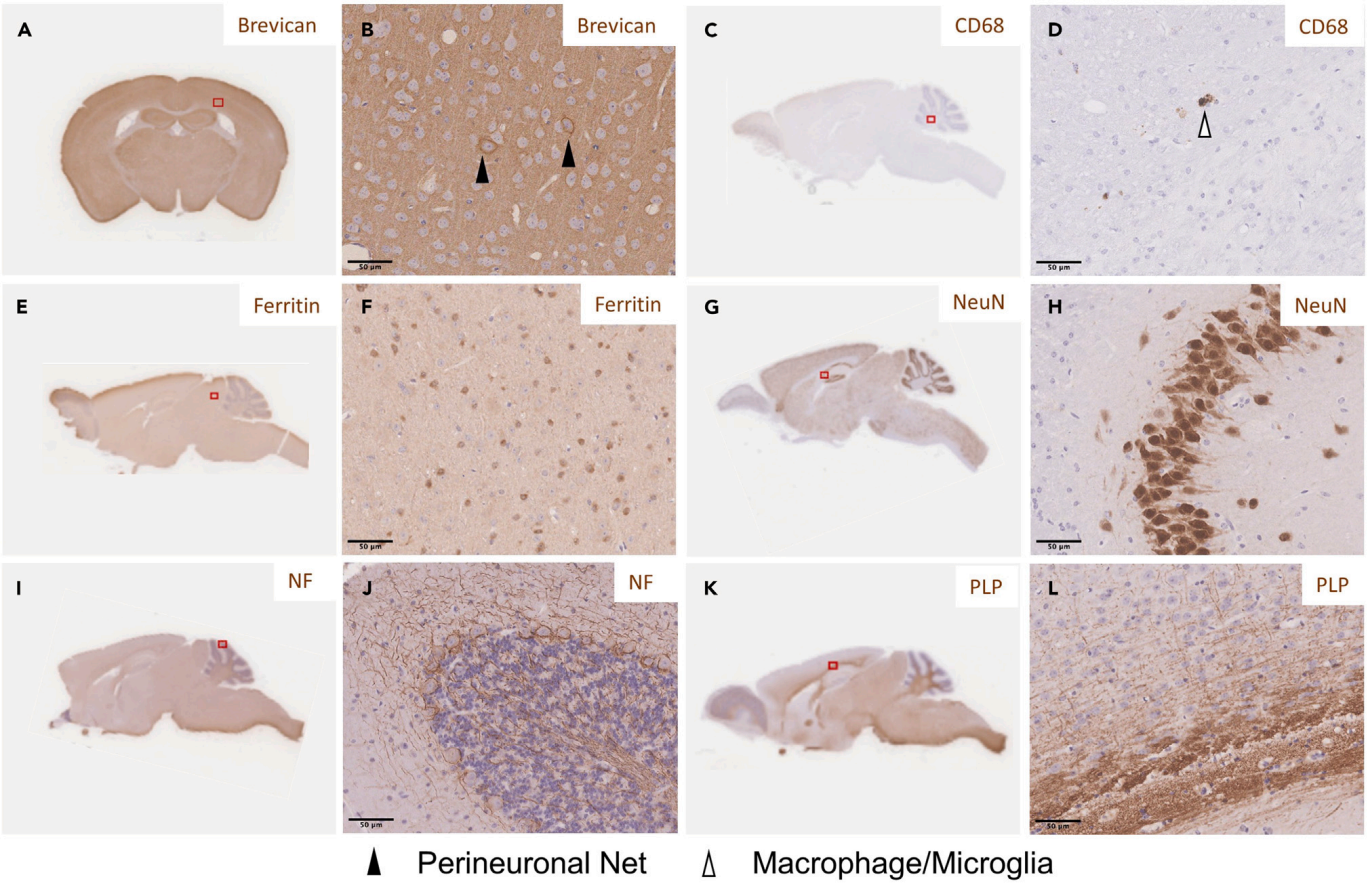
Expected outcome Representative images of Brevican (A, enlarged in B), CD68 (C, enlarged in D), Ferritin (E, enlarged in F), NeuN (G, enlarged in H), Neurofilament (I, enlarged in J) and PLP (K, enlarged in L) staining. Neurofilament (NF). Perineuronal nets (black arrowhead) and macrophages/microglia (white arrowhead). Scale bar is 50 μm. Red box indicates location of higher magnification panel.

Histology can provide anatomical ground truth to imaging data and tell us to what extent MRI signals reflect the underlying microstructure and elucidate the potential tissue constituents that may drive contrast in MR images. In this paper we present a protocol to improve the quality of adult mouse brain tissue that was stored in PBS, azide and Gadolinium based contrast agent at 4°C after fixation for an extended amount of time for *ex vivo* MRI. Prolonged storage in PBS can result in tissue swelling and defixation.^25^^,^^26^ The goal was to obtain stained slides with minimal folds, tears and changes in morphology to enable registration to MRI data. All steps from brain dissection to staining can affect the brain morphology in histological images. In particular, tissue processing can have a significant downstream impact, so it is important to ensure the correct schedule is chosen for the tissue size and type. Figure 8 shows a representative image for each tissue processing protocol (Table 4). Samples were sectioned and stained by the same operator using the same equipment and staining protocol. We found that using a standard^11^ tissue processing protocol resulted in tissue that was brittle and fragmented easily (Figures 8C and 8D). Furthermore, we observed more artefacts compared to the optimized protocol. Folds, wrinkles, and tears in the tissue and overexpansion of the section make registration more difficult (Figure 7G). The optimized protocol made it easier to achieve sections suitable for registration (Figures 8A and 8B; Figure 9). Our protocol was tested on samples stored after perfusion for up to 27 months with satisfactory histology results (Figure 9), which can be successfully registered to MRI data (Figure 7).

**Table 4:** Comparison of the standard and optimized tissue protocols

Step	Standard			Optimized		
	Reagent	Time (min)	Temperature (°C)	Reagent	Time (min)	Temperature (°C)
Rinsing	dH_2_O	<1	18‒22	dH_2_O	<1	18‒22
	dH_2_O	<1	18‒22	dH_2_O	<1	18‒22
Dehydration	70% Ethanol	60	18‒22	70% Ethanol	660	18‒22
	80% Ethanol	60	18‒22	80% Ethanol	45	18‒22
	95% Ethanol-I	60	18‒22	95% Ethanol-I	45	18‒22
	95% Ethanol-II	60	18‒22	95% Ethanol-II	45	18‒22
	100% Ethanol-I	30	18‒22	100% Ethanol-I	15	18‒22
	100% Ethanol-II	30	18‒22	100% Ethanol-II	15	18‒22
	100% Ethanol-III	30	18‒22	100% Ethanol-III	15	18‒22
	100% Ethanol-IV	30	18‒22	100% Ethanol-IV	30	18‒22
	100% Ethanol-V	30	18‒22	100% Ethanol-V	30	18‒22
	100% Ethanol-VI	30	18‒22	100% Ethanol-VI	30	18‒22
Clearing	CitroClear-I	60	18‒22	CitroClear-I	30	18‒22
	CitroClear-II	60	18‒22	CitroClear-II	45	18‒22
	CitroClear-III	60	18‒22	CitroClear-III	60	18‒22
Infiltration	Paraffin Wax-I	60	60	Paraffin Wax-I	30	60
	Paraffin Wax-II	60	60	Paraffin Wax-II	45	60
	Paraffin Wax-III	60	60	Paraffin Wax-III	60	60

Distilled water (dH_2_O).

## Limitations

The protocol here describes how to achieve good quality sections for histology after *ex vivo* MRI, therefore it may not be suitable for freshly prepared tissue. Long storage times impact the quality of the tissue and introduce artefacts. The tissue used in this protocol has been stored for up to approximately two years for MRI and the quality may have degraded. Furthermore, this tissue processing protocol may not be suitable for larger specimens. Generally, larger tissue samples will require a longer tissue processing protocol. Hurley, 2023^27^ describes a protocol for whole mouse tissue processing. However, the methods used to section, stain and register tissue described in this manuscript may also be generalized to larger tissue samples such as whole mouse brain.

Staining is best performed on freshly cut sections as long storage times, oxidation and exposure to light can reduce antigenicity of the tissue section.^14^^,^^28^ We recommend practicing sectioning tissue before attempting it on experimental tissue. Achieving sections without artefacts requires experience. It may be difficult to achieve serial sections with tissue that is particularly dry or brittle. Dependent upon study design, it may be necessary for all serial sections to share the same MRI voxel location.

Alignment between histology and MRI data can be difficult as samples may shrink during processing requiring correction during registration.^16^^,^^29^ In this study, an updated version of TIRL accounts for 3D deformations, high resolution, and small specimen size.^16^

## Troubleshooting

### Problem 1

Some common embedding problems (steps 11‒14).

### Potential solution

• Cracks in the paraffin blocks can be caused by the blocks cooling too quickly. Remelt the block and re-embed the tissue in the block.
• Hard material within the block causes scoring of tissue when sectioning. Remelt the block, remove the hard material and re-embed the tissue in the block.
• Overfilled mold results in the block sitting unevenly in the microtome. Use a blade to remove excess wax from around the block once it has solidified.
• The tissue is touching the edge of the mold. Either remelt and reposition the specimen within the block or use a larger embedding mold.

### Problem 2

Tissue is brittle or smells of solvent (step 15).

### Potential solution

• If the tissue is brittle or dry.
  ○ The tissue may be overprocessed. Soak the face of the block softening agents, such as Mollifex, and then in ice water. Adjust the section thickness. If the problem persists, reduce the processing time in subsequent samples.
  ○ If the sample is too small, it may become overprocessed. Reduce the tissue processing time for other samples.
• If the tissue smells of clearing agent or feels soft:
  ○ The Citroclear was not sufficiently displaced by the paraffin wax. Melt the block then place the tissue into fresh molten wax. Re-embed the tissue into a block.
  ○ The tissue may be under-processed due to the sample being too large or the schedule too short. Samples can be reprocessed using fresh reagents.

### Problem 3

The section shows small rough holes (chatter or moth-eaten artefact) (step 19).

### Potential solution

The block was trimmed too roughly/quickly. After trimming, polish the block by slowly removing a few thin sections then sections can be taken.

### Problem 4

Tissue section is highly wrinkled or compressed (step 23).

### Potential solution

• Gently remove wrinkles with a brush while the tissue is floating on the water bath.
• The wax may be too warm so it may be necessary to rechill the block.
• A blunt blade may cause the section to compress. Replace the blade.
• A too great clearance angle increases frictions as the blade passes which causes compression. Adjust the clearance angle.
• Add a non-standard flotation step. Transfer the section to room temperature 20% ethanol before transferring to the warm water bath. 20% ethanol has a lower surface tension than water, so it actively spreads the wrinkles out.
• Another cause may be a too cool water bath. Try increasing the temperature by a few degrees but note that increasing the temperature too much can cause overexpansion.
• Reduce the surface tension of the water in the water bath by adding a drop of alcohol or detergent.^13^

### Problem 5

Tissue sections fall off or the section lifts off the slide (step 31).

### Potential solution

• Use positively charged or coated histology slides.
• Ensure slides are completely dry before staining. Increase drying time if necessary.
• Increase baking time at 60°C.
• Alternative heat-mediated retrieval equipment such as a water bath, pressure cooker or steamer could be used; however, we did not test these for the current protocol.

### Problem 6

Strong background, non-specific or weak target staining (step 36).

### Potential solution

• If the slides are over stained:
  ○ Add extra washing steps to the immunohistochemistry protocol to ensure the primary and secondary antibodies are being removed.
  ○ Reduce the concentration of the primary antibody.
  ○ Try using an alternative blocking reagent. It is recommended to match the host species of the secondary antibody when blocking with normal serum. Alternatively, serum-free protein blocks can also be used.
  ○ When repeating the stain include a “no primary” control to identify if the background is caused by nonspecific binding of a reagent that is not the primary antibody.^30^
• If the slides show non-specific staining:
  ○ Reduce the concentration of the antibody.
  ○ Try different blocking serums.
  ○ Try an alternative antibody.
• If the slides are weakly stained:
  ○ Deparaffinization may be inadequate. Increase the time in xylene and use fresh xylene.
  ○ Antibody concentration may be too low. Increase the antibody concentration.
  ○ Heat retrieval may be insufficient. Test different heat mediated antigen retrieval conditions, e.g., Citrate pH 6 or Tris-EDTA pH 9. Do not use Tris-EDTA pH 9 in the microwave.
  ○ Include a positive control from a tissue known to express the antigen of interest to verify the viability of the antibody.^30^

## Resource availability

### Lead contact

Further information and requests for resources and reagents should be directed to and will be fulfilled by the lead contact, Adele Smart (adele.smart@ndcn.ox.ac.uk).

### Materials availability

This study did not generate new unique reagents.

## Data Availability

This study did not generate new unique codes.
